# A Theoretical Study on the Efficacy and Mechanism of Combined YAP-1 and PARP-1 Inhibitors in the Treatment of Glioblastoma Multiforme Using Peruvian Maca *Lepidium meyenii*

**DOI:** 10.3390/cimb47010040

**Published:** 2025-01-09

**Authors:** Albert Gabriel Turpo-Peqqueña, Sebastian Luna-Prado, Renato Javier Valencia-Arce, Fabio Leonardo Del-Carpio-Carrazco, Badhin Gómez

**Affiliations:** 1 Centro de Investigación en Ingeniería Molecular–CIIM, Universidad Católica de Santa María, Urb. San José s/n, Umacollo, Arequipa 04013, Peru; albert.turpo@ucsm.edu.pe (A.G.T.-P.); sebastian.luna@ucsm.edu.pe (S.L.-P.); 71505948@ucsm.edu.pe (R.J.V.-A.); 70666250@ucsm.edu.pe (F.L.D.-C.-C.); 2 Facultad de Medicina Humana, Universidad Católica de Santa María, Urb. San José s/n, Umacollo, Arequipa 04013, Peru; 3 Facultad de Biología, Universidad Nacional de San Agustín, Av. Alcides Carrión s/n, Arequipa 04001, Peru; 4 Facultad de Ciencias Farmacéuticas, Bioquímicas y Biotecnológicas, Universidad Católica de Santa María, Urb. San José s/n, Umacollo, Arequipa 04013, Peru

**Keywords:** glioblastoma multiforme, Neuro-oncology, Neurosurgery, Molecular Mechanics, molecular docking, molecular dynamics simulation, PARP-1 inhibitors, YAP-1 inhibitors, *Lepidium meyenii*

## Abstract

Glioblastoma multiforme (GBM) is one of the most aggressive and treatment-resistant forms of brain cancer. Current therapeutic strategies, including surgery, chemotherapy, and radiotherapy, often fail due to the tumor’s ability to develop resistance. The proteins YAP-1 (Yes-associated protein 1) and PARP-1 (Poly-(ADP-ribose)–polymerase-1) have been implicated in this resistance, playing crucial roles in cell proliferation and DNA repair mechanisms, respectively. This study explored the inhibitory potential of natural compounds from *Lepidium meyenii* (Peruvian Maca) on the YAP-1 and PARP-1 protein systems to develop novel therapeutic strategies for GBM. By molecular dynamics simulations, we identified N-(3-Methoxybenzyl)-(9Z,12Z,15Z)- octadecatrienamide (DK5) as the most promising natural inhibitor for PARP-1 and stearic acid (GK4) for YAP-1. Although synthetic inhibitors, such as Olaparib (ODK) for PARP-1 and Verteporfin (VER) for YAP-1, only VER was superior to the naturally occurring molecule and proved a promising alternative. In conclusion, natural compounds from *Lepidium meyenii* (Peruvian Maca) offer a potentially innovative approach to improve GBM treatment, complementing existing therapies with their inhibitory action on PARP-1 and YAP-1.

## 1. Introduction

In recent decades, cancer has become a severe public health problem [1]. Among the various types of cancer, Glioblastoma Multiforme (GBM) is the most aggressive type of cancer in adults [2] and the most lethal form of primary intracranial cancer [3], mainly due to its invasive nature, complex location, malignancy, rapid progression, and high resistance to conventional treatments [4,5]. Despite current advances in the fields of Surgery, Chemotherapy, and Radiotherapy [6], the treatments used have little efficacy [7], which is reflected in a low average overall survival rate, giving the patient a life expectancy of only 15 months [8]. It is due to the difficulty in performing a complete surgical resection due to a high recurrence rate [9], diffuse infiltration [10], molecular heterogeneity [11], and the ability to develop resistance to radiotherapy and chemotherapy [12]. Therefore, GBM currently represents a major therapeutic challenge [13] in Neurooncology and Neurosurgery.

One of the essential reasons for treatment resistance in GBM is related to the overexpression of critical proteins (Yes-1 Associated Transcriptional Regulator (YAP-1) [14] and Poly-(ADP-ribose) polymerase-1 (PARP-1) [15,16]) that regulate cellular homeostasis. We have found in the literature reports that YAP-1 plays a vital role in cell proliferation [17], while PARP-1 in DNA repair [18]. Moreover, overexpression not only contributes to the ineffectiveness of chemotherapy and radiotherapy [14] but also to tumor aggressiveness, which translates into poor patient survival [19,20,21,22]. In this context, there is an urgent need to find new approaches to improve the efficacy of conventional treatment and increase patient survival. YAP-1 is one of the most essential transcriptional coactivators in the HIPPO signaling pathway [23]. In the cellular environment, the MST-1/2 and LATS-1/2 kinases activate the phosphorylation of YAP-1. This phosphorylation facilitates the binding to 14-3-3 proteins, which promotes retention in the cytoplasm and its degradation. However, when the pathway is inactive, YAP-1 is dephosphorylated, becoming free and translocated to the nucleus, where it interacts with TEAD, regulating cell growth and organogenesis [24,25,26]. In GBM, an overexpression of YAP-1 has been observed [20], and currently, therapies directed explicitly against this protein are scarce [27]. Available drugs, such as Verteporfin, inhibit YAP-1 indirectly [28]. Although the FDA initially approved Verteporfin for macular degeneration, it has also been studied as part of drug repurposing for GBM [29] as it inhibits YAP-1 interaction with TEAD [30]. However, its efficacy remains limited in GBM. Therefore, studies are needed to understand better the role of YAP-1 in gliomagenesis, and directly inhibiting YAP-1 would be a novel approach in personalized medicine to treat GBM.

On the other hand, PARP-1 is responsible for 90% of the activity of ADP-ribosyl transferase, whose enzymatic action is known as PARylation in human cells, regardless of whether the cells are normal or malignant [31]. PARP-1 plays a crucial role in DNA repair; in the event of damage, PARP-1 rapidly binds to the affected DNA, catalyzing the decomposition of NAD+ and generating poly-ADP-ribose (PAR). PAR recruits repair enzymes such as XRCC1 (X-ray repair cross-complementing 1) and DNA ligase III, which perform base excision repair (BER), thus correcting damaged bases [32]. PARP-1 is overexpressed in various types of cancer, including GBM, suggesting its pro-tumorigenic role. PARP-1 overexpression has been linked to disease progression, as this enzyme is essential for tumor cell survival in response to conventional radiotherapy and chemotherapy. PARP-1 overactivation allows malignant cells to efficiently repair DNA damage, facilitating their continued proliferation and contributing to resistance [33,34,35]. PARP-1 inhibitors such as Olaparib [36] and Veliparib [37] have been combined with Temozolomide to improve GBM treatment outcomes. Therefore, the combination with other therapies represents a promising avenue of research for treating GBM.

*Lepidium meyenii*, commonly known as Peruvian Maca, an annual or biennial plant of the Brassicaceae family, grows mainly at 4000 m above sea level in the Andean region of Peru [38,39]. So far, more than 100 active secondary metabolites have been found in Maca, including alkaloids, phytosterols, flavonols, glucosinolates, hydantoins, isothiocyanates, macaenes, and macamides. However, only 10% of these secondary metabolites present pharmacological activity [40,41]; these include reproductive health promotion, memory enhancement, antidepressant, neuroprotection, antioxidation, antifatigue, anticancer, hepatoprotection, antiosteoporosis, antidysmnesia, immunomodulation, anti-inflammatory and skin protection [42,43]. Since both YAP-1 and PARP-1 play critical roles in GBM progression and resistance, simultaneously inhibiting these two proteins could offer a synergistic strategy to overcome therapeutic resistance and improve clinical outcomes. In this context, naturally occurring metabolites, such as those in *Lepidium meyenii* [44,45,46], represent a potential source of compounds with YAP-1 and PARP-1 inhibitory properties.

Computational studies offer an innovative avenue for the development of personalized therapies by using naturally occurring molecules that possess properties of activators or inhibitors of protein systems involved in neurological processes, one of the most significant challenges in modern neurooncology [47]. This research seeks to evaluate the potential properties of some *Lepidium meyenii* metabolites with YAP-1 and PARP-1 to identify compounds with therapeutic potential in treating GBM.

## 2. Computational Details

Obtaining the three-dimensional structures of the YAP-1 and PARP-1 enzymes was essential to start the computational simulation process. For the case of YAP-1, its molecular structure was found in the PDBe and PDBe-KB databases [48,49], which list different structures from multiple experimental procedures with other characteristics such as sequence size, mutations, quality, etc. Considering the above, we obtained the enzyme’s structure with the identification code 6GEI [50]. Due to the empirical nature of the origin of the structure, it was necessary to eliminate the crystallized water molecules and complete the protein structure with the AlphaFold tool [51,52,53] using the Chimera X program [54]. We used these tools because none of the total structures listed in the databases contains more than 60 residues of the 504 YAP-1 should have. As for PARP-1, it was downloaded from the PDB database [55,56] with the code 4DQY [57], and we followed the same cleaning and completion process. However, since the AlphaFold tool did not consider the Zinc atoms, we manually added the Zinc fingers of the enzyme. On the other hand, we obtained the molecular structure of these naturally occurring interacting compounds through the PubChem database [58]. In the studies performed with the PARP-1 protein, the metabolites N-benzylhexadecanamide (MC1) [59], N-benzyl-5-oxo-6E,8E-octadecadienamide (MC2) [60] and N-(3-Methoxybenzyl)-(9Z,12Z,15Z)-octadecatrienamide (DK5) [61] were used. Also, in the investigations with the YAP-1 protein, the metabolites stearic acid (GK4) [62], pentahomomethionine (GK1) [63], phytosphingosine [64], N-benzylpentadecanamide (GK3) [65] and N-benzyl-5-oxo-6E,8E-octadecadienamide (MC2) [60] were used. In addition, for comparison, synthetic ligands such as Veliparib (VDK) [66] and Olaparib (ODK) [67], both used in the studies with the PARP-1 protein, as well as Verteporfin (VER) [68], which we used with the YAP-1 protein, were also included. Unlike the other compounds, we obtained the Verteporfin from the ChemSpider database [69].

We used GROMACS [70,71] to optimize and balance the complex structures of the YAP-1 and PARP-1 enzymes. We also used a cubic box to center the proteins we solvated with water molecules. Additionally, we neutralized the systems with chloride and sodium ions. We minimized the forces between the atoms; then, we introduced the thermostat at a constant temperature of 309.65 K (NVT) for ten ns and a constant pressure of 1 bar (NPT) for 500 ns. In the case of the ligands, we generated their topology in the online server LigParGen [72,73,74] to then optimize coordinates with the semiempirical method AM1 [75], and later, the Hirshfeld charges were calculated with the CAM-B3LYP functional [76], using the Gaussian 16 program [77].

After optimizing all the structures that will be part of the simulation process, we preliminarily analyzed the most probable druggable pockets of the two proteins under study using the CavityPlus server [78]. Next, we perform blind molecular docking, assembling the protein-ligand complexes. Using the AutoDock Vina tool [79,80], we performed two thousand tests with each of the ligands, in which we placed them using a stochastic method on the surface of the proteins, selecting the tests that present a higher interaction energy in kcal/mol.

Once we built the protein-ligand complexes, the molecular dynamics were simulated in an isothermal and isobaric system for 10 and 100 nanoseconds, respectively, with the GROMACS program. We analyzed the trajectories of this simulation using the RMSD, RMSF, RG, and HB graphs to determine the stability, variability, and compaction of the protein structure over the simulated time; we made these graphs with GNUplot [81]. Finally, it was necessary to analyze the nature of the interaction between the proteins and the interacting compounds, for which we used PDBsum [82], an online server, to determine the bonds and contacts generated in the complex after the molecular dynamics. MMPBSA [83,84] to calculate the energies involved in the binding of the complex, APBS [85] to visualize the changes in the surface charge of the proteins that may be a consequence of the protein-ligand interaction and ADMETlab 3.0 [86] to evaluate the pharmacological properties of the compounds.

## 3. Results and Discussion

The PARP-1 structure obtained from the PDB database (ID: 4DQY) contained six chains, a DNA segment, two zinc atoms per trimer, and ethylene glycol molecules used in the crystallization. The Chimera X software removed structures foreign to PARP-1, such as DNA and ethylene glycol. We observed that some residues were missing, completed with the Alphafold server. The PARP-1 structure (ID: 4DQY), composed of 1014 residues and two zinc atoms, was solvated in a cubic box of 13.4414 nm per side using the TIP3P water model. We neutralized the system with 26 chlorine atoms because it had a total charge of +26, and we added 454 salt ions (Na+ and Cl−) to reach a concentration of 0.15 M. In total, we included 74,482 water molecules.

**Figure 1:**
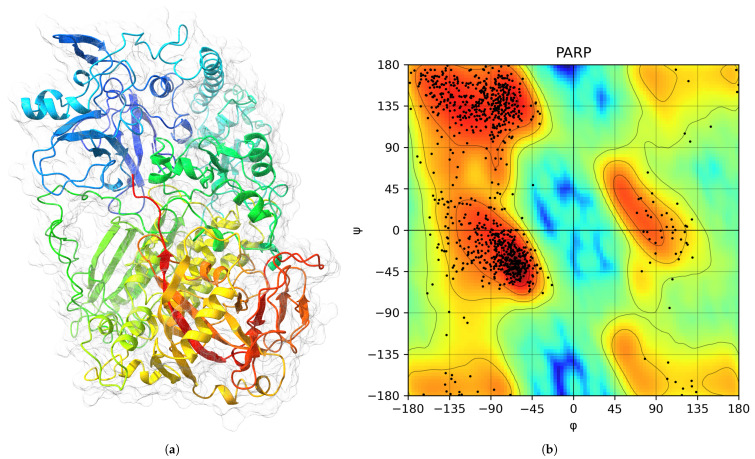
(**a**) Structure of PARP1 in rainbow scale colors, ID: 4DQY and (**b**) A Ramachandran diagram analysis of PARP-1 after a 500 ns simulation in a rainbow scale: red indicates more accumulation, and blue indicates less accumulation of residuals.

We minimized the forces on the system with the steepest descent algorithm to a 1.0 kJ/mol tolerance. Subsequently, we introduced the thermostat at a temperature of 309.65 K for 10 ns. We introduced the barostat at a pressure of 1 bar for a period of 10 ns, and after this, we performed a molecular dynamics simulation for some time of 500 ns, with steps of 1 fs (see Figure 1a).

The final structure of PARP-1 was analyzed after molecular dynamics simulation by a Ramachandran diagram (Figure 1b) to assess the natural conformation of the structure. Of the 897 residues we analyzed 748 (83.4%) it is in favored regions, 136 (15.2%) in allowed regions, and 11 (1.2%) in generously allowed regions, which amounts to 99.8% of the residues in acceptable conformations. We found only two residues (0.2%) in the disfavored areas. This analysis excluded glycines, prolines, and preprolines.

**Figure 2:**
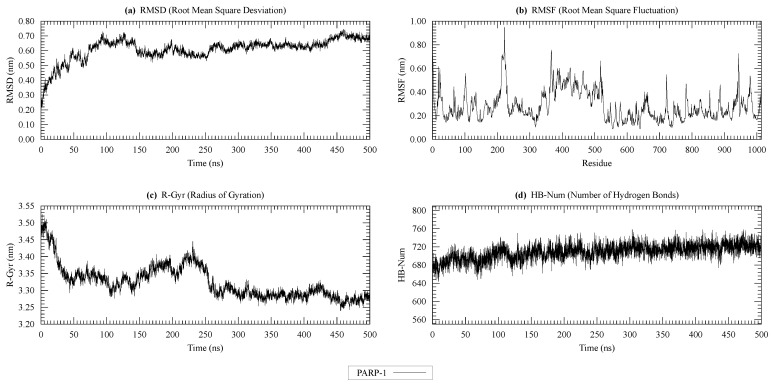
PARP-1 stabilization properties after a 500 ns molecular dynamics simulation: (**a**) RMSD; (**b**) RMSF; (**c**) Radius of gyration; (**d**) Number of hydrogen bonds.

By analyzing the root mean square deviation of the distances, RMSD (see Figure 2a), we observe that the PARP-1 (4DQY) system reached equilibrium after 250 ns due to a variation of 0.2 nm, which follows empirical observations. In the graph of the root mean square deviation of the fluctuations of the residues (see Figure 2b), we observe that the regions with the most significant movement are between residues 200–240, 360–530, and 920–990. Regarding the radius of gyration (Figure 2c), the system shows a compaction process from the beginning, with a temporal expansion between 100 ns and 250 ns. The x-axis contributes more to the expansion, while the y-axis favors the compaction.

The cleaned YAP-1 structure, containing 504 residues, was placed in a triclinic box of dimensions 481.564 nm per side, and we carried out the solvation in the volume with the TIP3P water model. Next, we neutralized twenty-six positive (+26) charges using chlorine atoms, indicating a total charge of the system due to the physiological pH. We added sodium and chlorine salt ions, corresponding to a concentration of 0.15 M. This resulted in thirteen thousand five hundred nine (13,509) water molecules in the box. With this system, we minimize the forces using the steepest descent algorithm, with an energy tolerance of 1.0 kJ/mol. After this step, we introduced the temperature of 309.65 K for ten ns, and then the pressure of 1 bar was introduced for ten ns. We used the resulting files to start the molecular dynamics simulation with a trajectory of 500 ns in steps of 1 fs (Figure 1a).

**Figure 3:**
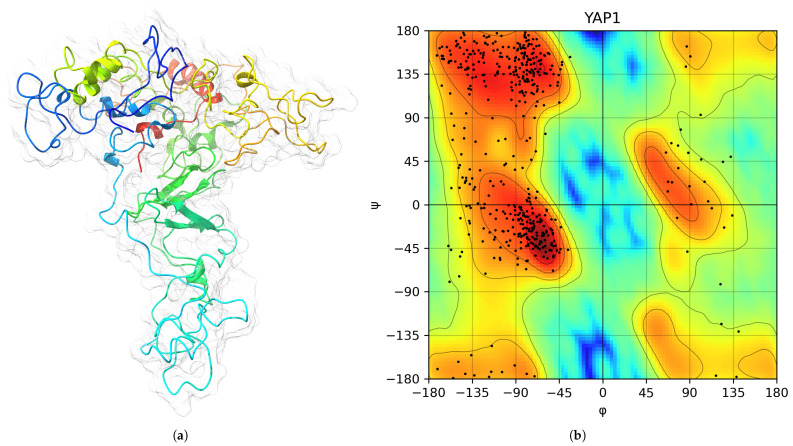
(**a**) Structure of YAP-1 in rainbow scale colors, ID: 6GEI and (**b**) A Ramachandran diagram analysis of YAP-1 after a 500 ns simulation in a rainbow scale: red indicates more accumulation, and blue indicates less accumulation of residuals.

We performed a Ramachandran diagram analysis (Figure 3b) to assess the structure’s natural conformation. We observed that 311 residues (76.4%) are in favored regions, 82 (20.1%) in additional allowed regions, and 7 (1.7%) in generously allowed regions, accounting for 98.2% of the residues. We found only seven residues (1.7%) in the disfavored areas. This analysis excluded glycines and prolines, analyzing a total of 504 residues.

**Figure 4:**
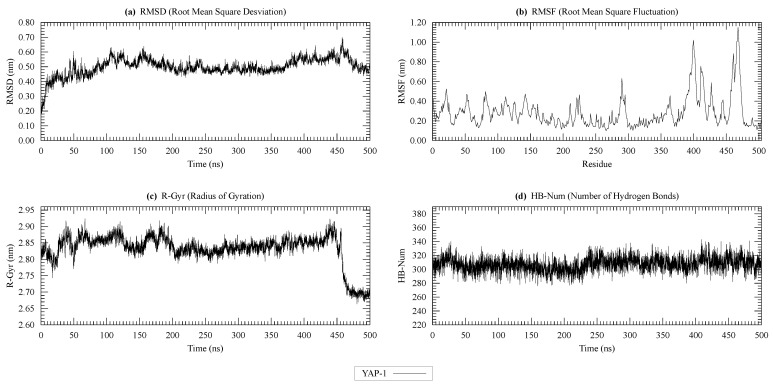
Stabilization properties of YAP-1 after a 500 ns molecular dynamics simulation: (**a**) RMSD; (**b**) RMSF; (**c**) Radius of gyration; (**d**) Number of hydrogen bonds.

When analyzing the root mean square deviation of distances (RMSD) for the YAP-1 system (Figure 4a), we observe that it reaches equilibrium starting from 100 ns, maintaining a reasonable stability with a delta of 0.2 nm up to 500 ns. In the root means square deviation plot of residue fluctuations (RMSF) (Figure 4b), the regions with the most significant movement are located between residues 250–300 and 400–470. Regarding the radius of gyration (Rg) (Figure 4c), the YAP-1 protein experiences initial compaction.

**Figure 5:**
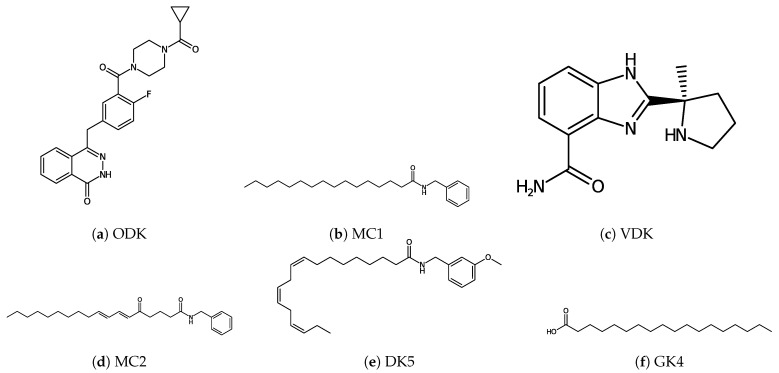
Two-dimensional structure of naturally occurring and synthetic PARP-1 inhibitors.

In Figure 5, we present the structures of naturally occurring and synthetic compounds found in the databases with potential PARP-1 inhibitory activity in a two-dimensional (2D) representation.

**Figure 6:**
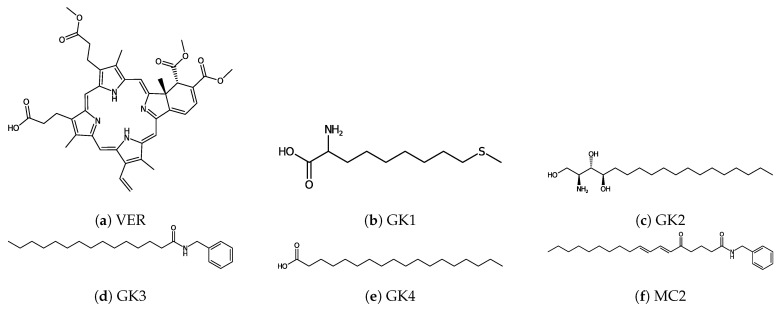
Two-dimensional structure of naturally occurring and synthetic YAP-1 inhibitors.

Similarly, Figure 6 presents the structures of both naturally occurring and synthetic compounds found in the databases with potential YAP-1 inhibitory activity in a two-dimensional (2D) representation.

The druggable pockets were analyzed using the CavityPlus server for PARP-1 and YAP-1. Table 1 presents the results obtained for the six best druggable pockets. For PARP-1, the highest druggable site has a value of 8047 and the lowest a value of 2651, in arbitrary druggability units, while for YAP-1, the highest druggable site has a value of 2340 and the lowest has a value of −129, in arbitrary druggability units. As we can see, the higher the druggability value, the higher the probability that an interaction with a molecule occurs in that region. However, if the value is negative, the likelihood of interaction is much lower. However, we must remember that we calculated this druggability on a static protein; this may vary because the system is in motion.

**Figure 7:**
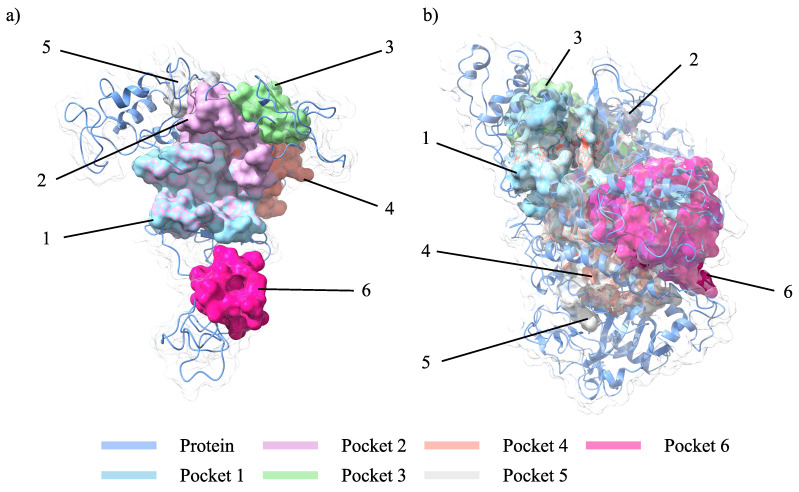
Druggable pockets of (**a**) PARP-1 y (**b**) YAP-1.

In Figure 7, we can observe the druggable pockets calculated using the CavityPlus server. The pockets are identified by color and numbered according to Table 1.

Next, we used the computational package Autodock Vina tools, with which we docked the natural and synthetic compounds with the stabilized structure of PARP-1 and YAP-1. For each compound, 2000 events were carried out with a total of 20,000 dockings, selecting the system with the lowest energy for each case (see Table 2). We noted that the energy values provided by AutoDock Vina are referential because the force field considers geometric parameters derived from Van der Waals volumes.

Analyzing the results of the binding energies for the complexes with PARP-1, we can observe that the synthetic inhibitors present better interaction energies. ODK is the compound with the best interaction energy within this group, with a value of −11.13 kcal/mol, followed by VDK with −9.34 kcal/mol. In comparison, the best natural origin compound is DK5, with an interaction energy of −8.99 kcal/mol, followed by MC2 with −8.73 kcal/mol and MC1 with −8.10 kcal/mol. The difference in binding energy between the best synthetic compound (ODK) and the best natural compound (GK5) is 2.14 kcal/mol, which indicates a higher affinity of the artificial compound for PARP-1. However, the energy difference between the natural inhibitors is relatively small, suggesting they could also be good candidates.

Regarding the inhibitors interacting with YAP-1, the synthetic inhibitor VER shows the best binding energy, with a value of −10.40 kcal/mol, positioning itself as the compound with the highest affinity. On the other hand, the best compound of natural origin is MC2, with an interaction energy of −9.16 kcal/mol, followed by GK3 with −7.92 kcal/mol. The difference between VER and MC2 is 1.24 kcal/mol, indicating that, although the synthetic compound has a better affinity, the natural compounds also interact well with the YAP-1 protein. The interaction energies obtained from any docking method are only referential because it is a probabilistic method, and the force fields used are completely simplified to show the terms due to non-binding interactions, so we performed an additional molecular dynamics simulation using deterministic methods to identify the molecular interaction processes correctly.

Thus, we performed the molecular dynamics simulation under the same temperature, pressure, and salt ion concentration conditions for each of the selected systems for a 100 ns trajectory. The results obtained were analyzed, mainly the interaction energy of the last ten ns of each system, through the MMPBSA approximation. The tables show the different energy components into which we decomposed the free energy of interaction.

In Table 3, we show the free energies of interaction for PARP-1 with the different ligands, which present a scale in the free energy of interaction of DK5>ODK>MC1>MC2≈GK4>VDK, the value for DK5 is −93.89±6.79 kcal/mol, while that for VDK is −68.15±6.74 kcal/mol. When we analyze the contribution to the free energy by Van der Walls interactions, we find that MC2≈ODK>DK5>MC1>GK4>VDK, the value for MC2 is −55.00±2.03 kcal/mol, while that for VDK is −41.51±2.33 kcal/mol. In the case of the contribution of electrostatic interactions to the free energy of interaction, we know that those that are above the significant error of the energy (2 kcal/mol) are important; therefore, when looking at Table 2, we find that this contribution has the following order ODK>GK4≈MC1, the value for ODK is −9.91±2.96 kcal/mol, while for MC1 it is −3.17±3.30 kcal/mol. Likewise, when we analyze the contribution of the interactions of polar solvents with the interacting systems towards the free energy of interaction, we find that MC2>ODK>DK5≈DK4>MC1>VDK, the value for MC2 is 29.11±3.57 kcal/mol. At the same time, for VDK, it is 16.89±4.56 kcal/mol. Additionally, the contribution to the free energy of interaction by the solvent-accessible surface area gives us the following order DK5≈MC2≈GK4≈MC1≈ODK>VDK; the value for DK5 is −5.81±0.25 kcal/mol. At the same time, for VDK, it is −3.76±0; finally, the contribution of the solvent-accessible volume towards the free energy of interaction has the following order DK5>MC2>MC1>GK4>ODK>VDK, the value of DK5 is −54.72±5.35 kcal/mol, while for VDK it is −37.99±3.08 kcal/mol. We can observe that both the Van der Waals energy and the solvent-accessible volume increase the binding free energy for DK5, above that of the other secondary metabolites and over that of the two drugs used as controls. It is essential to highlight that we have found that DK5 is the metabolite that presents a potential inhibitory activity against PARP-1.

The results for YAP−1 with the selected ligands we presented in Table 4; if we consider the overall interaction free energy, we obtained the following order: VER>GK4>MC2>GK3>GK2>GK1, the value for VER is −121.37±10.75 kcal/mol and while for GK1 it is −62.36±10.28 kcal/mol. When we analyze the various contributions to the interaction-free energy, we find that the Van der Waals terms contributing to the interaction energy present the following order VER>MC2>GK4>GK3>GK2>GK1, presenting the value for VER of −64.28±4.50 kcal/mol, while for GK1 it is −31.33±4.04 kcal/mol; Likewise, in the case of contributions due to Coulombic interactions, they presented the following order VER>GK3>GK4>MC2≈GK1, the value for VER being −9.54±4.75 kcal/mol, and for GK1−3.14±4.41 kcal/mol. We did not consider GK2 because its value is below the calculation error.

Furthermore, in the case of the contribution of interactions due to polar solvents, which, in the cases of our present study, are unfavorable, presenting the order MC2>GK3≈VER>GK2>GK4>GK1, the value for MC2 is 24.57±2.35 kcal/mol while for GK1 it is 10.23±2.40 kcal/mol; In contrast, the contribution of the solvent-accessible surface area to the free energy of interaction is VER>MC2≈GK3≈GK4>GK2>GK1, the value for VER is −7.53±0.41 kcal/mol while for GK1 it is −3.56±0.27 kcal/mol; finally, the contribution of the solvent-accessible volume to the free energy of interaction presents the order VER>MC2>GK4>GK3>GK2>GK1, the value for VER being −64.59±7.26 kcal/mol. In comparison, GK1 is −34.55±4.92 kcal/mol. In the case of YAP−1. We can observe that the Van der Waals energy, solvent-accessible volume energy, and polar solvent energy increase the binding free energy of the VER drug above that of the secondary metabolites. The VER metabolite presents favorable characteristics that inhibit the YAP−1 protein system.

**Figure 8:**
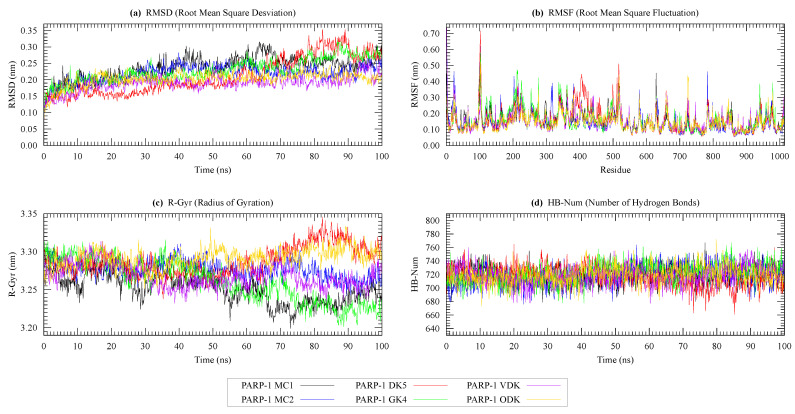
PARP-1 stabilization properties with inhibitors after a 100 ns molecular dynamics simulation: (**a**) RMSD; (**b**) RMSF; (**c**) Radius of gyration; (**d**) Number of hydrogen bonds.

When we analyze the molecular dynamics simulation results for PARP-1 complexed with six molecules, we show the results for a 100 ns trajectory in Figure 8. The RMSD analysis (Figure 8a) shows that most complexes maintain a relatively stable structure during the trajectory. However, some, such as the PARP1-VDK complex, exhibit slight deviations along the trajectory, which indicate minor conformational modifications. On the other hand, the PARP1-MC1 complex presents a consistency in its trajectory. In Figure 8b, we show that the residual fluctuations (RMSF) are more pronounced in certain regions, with residues close to 100, from 380 to 420, and 500 to 520; this suggests a certain local flexibility that does not compromise the system’s structural integrity. The radius of gyration (R-gym), shown in Figure 8c, shows that the packing of the structures remains relatively constant, with no signs of significant expansion during the simulation, reflecting good compaction, except in the cases of DK5 and ODK from 75 ns onwards. Furthermore, the hydrogen bonds, analyzed in Figure 8d, remain generally constant throughout the trajectory, indicating that hydrogen interactions do not undergo significant changes and contribute to maintaining the secondary structure during the 100 ns.

**Figure 9:**
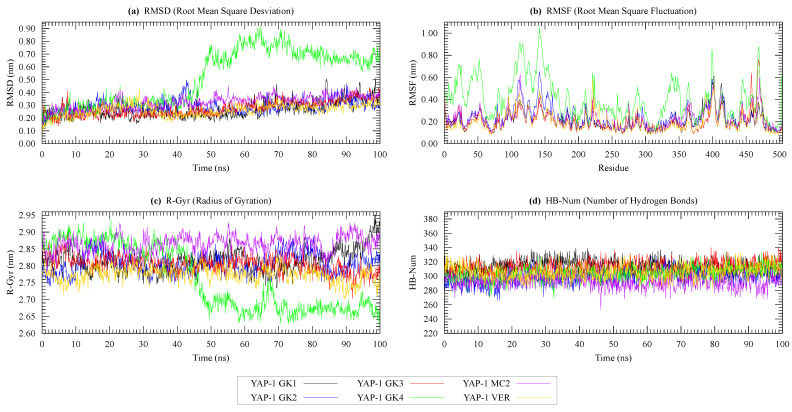
Stabilization properties of YAP-1 with the inhibitors after a 100 ns molecular dynamics simulation: (**a**) RMSD; (**b**) RMSF; (**c**) Radius of gyration; (**d**) Number of hydrogen bonds.

For the YAP-1 complexes with six molecules of natural and synthetic origin, we present the molecular dynamics simulation results in Figure 9. When analyzing the RMSD (Figure 9a), the values are much more variable than in the PARP complexes, especially in the case of the YAP1-GK4 complex, which at 43 ns undergoes a significant structural variation until approximately 74 ns, after which it tends to stabilize. On the other hand, the YAP1-GK2 complex system remains more stable during the trajectory, which indicates an apparent better affinity between the inhibitor and the protein. In Figure 9b, we show the variability of the fluctuations per residue (RMSF), where we can identify flexible regions in different protein parts. Still, it is essential to mention that in the case of GK4, the fluctuations are presented in the same areas but with a higher incidence. Looking at the R-gym in Figure 9c, we see that it remains relatively constant in most systems, suggesting that the structures remain or maintain their secondary structure, but in the case of GK4, we observe that it becomes even more compact after 43 ns, suggesting that the systems are gaining secondary structures in the protein. Finally, in Figure 9d, we show that when analyzing the hydrogen bonds, these are preserved without essential changes along the trajectory, indicating that these interactions do not play a relevant role in the system’s stability during the 100 ns of the molecular dynamics simulation.

It is essential to highlight that for PARP-1, the molecules that interact best are ODK and DK5, both have been located in pocket two according to Table 1, while for YAP-1, the druggable pockets for the two molecules favored by the free energy of interaction are pocket one for VER and pocket six for GK4 according to Table 1, we must emphasize that the druggability given by the CavityPlus service is referential because they are static. In contrast, in the case of the final location, both the proteins and the interacting molecules are in motion. Therefore, we located DK4 in a site of lower static druggability, which increases its affinity when the system is in motion.

When analyzing the properties of the final structure of the molecular dynamic’s simulation, we have only considered the synthetic and natural structures with the best interaction-free energy; for them, we have considered the Ramachandran diagram, the approximation of the electrostatic potential through APBS, and the affinity of some residues with the selected molecules.

**Figure 10:**
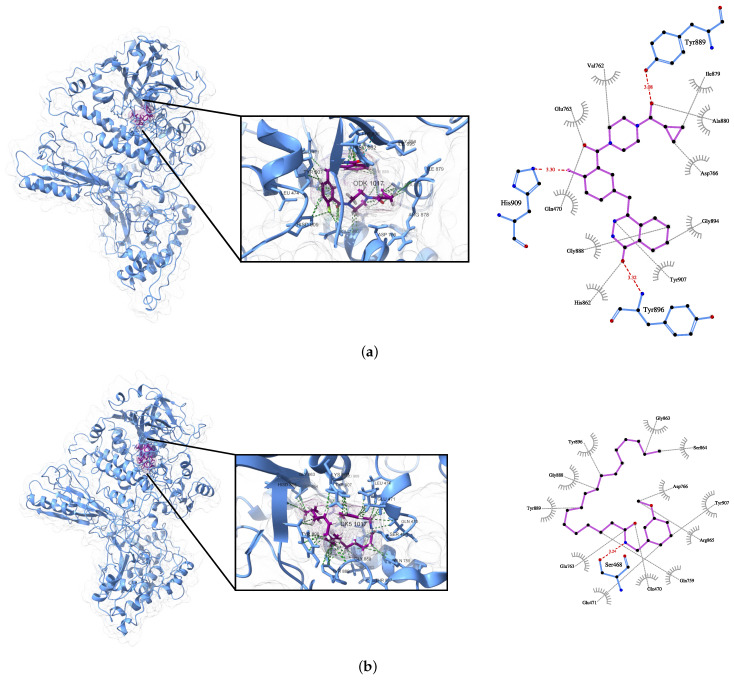
Structure of PARP-1 interacting with molecules: (**a**) ODK and (**b**) DK5.

In the case of PARP-1, among the molecules of synthetic origin, ODK presents the best free energy of interaction, and DK5 is the molecule of natural origin with the best free energy of interaction, as shown in Figure 10.

**Figure 11:**
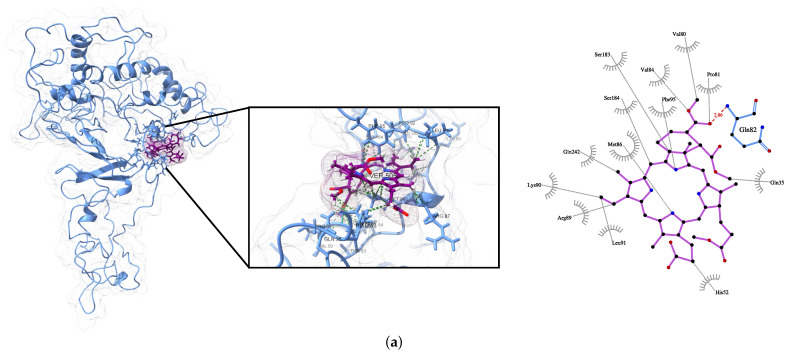

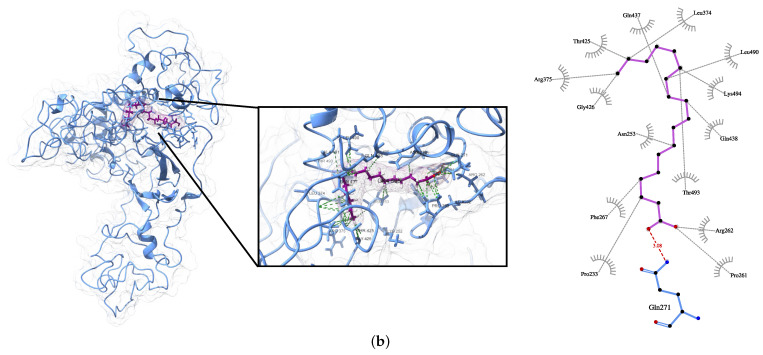
Structure of YAP-1 interacting with the molecules (**a**) VER (**b**) GK4.

When analyzing with the LigPlot server for PARP-1, in the case of ODK, it was possible to determine three hydrogen bridge bonds with the residues Tyr-889, Tyr-896, and His-909. Additionally, fifty-six non-binding interactions were found: Gln-470, Tyr-710, Val-762, Glu-763, Asp-766, His-862, Ile-879, Ala-880, Gly-888, Tyr-889, Gly-894, Tyr-896, Tyr-907 and His-909; varying the number of interacting centers per residue, being Tyr-896 the residue with the highest number of interactions. When we performed the analysis for DK5, we obtained a total of one hydrogen bond with the Ser-468 residue and thirty-seven non-bonding interactions: Ser-468, Gln-470, Glu-471, Gln-759, Glu-763, Asp-766, Gly-863, Ser-864, Arg-865, Gly-888, Tyr-889, Tyr-896 and Tyr-907; varying the number of interacting centers per residue, with Tyr-907 presenting the highest number of interactions. When we analyze the results for YAP-1 (See Figure 11), the VER and GK4 molecules present the lowest free energies of interaction. Thus, for VER, it has been found that it presents a hydrogen bond with the residue Gln-82, while the non-bonding interactions found are in several forty-six, between VER and the residues Gln-35, His-52, Val-80, Pro-81, Gln-82, Val-84, Met-86, Arg-89, Lys-90, Leu-91, Phe-95, Ser-183, and Gln-242, the interactions are varied in number with the residues, being Met-86 the one that presented the highest number of interactions.

In the case of GK4 with YAP-1, we did not find hydrogen bonds between the molecule and the receptor. Still, a total of thirty non-binding interactions were determined with the residues Pro-233, Asn-253, Pro-261, Arg-262, Phe-267, Gln-271, Leu-374, Arg-375, Thr-425, Gly-426, Gln-437, Gln-438, Leu-490, Thr-493, and Lys-494, presenting in some cases more than one interaction with the residues, being the residue Pro-233 the one that offered the highest number of interactions.

**Figure 12:**
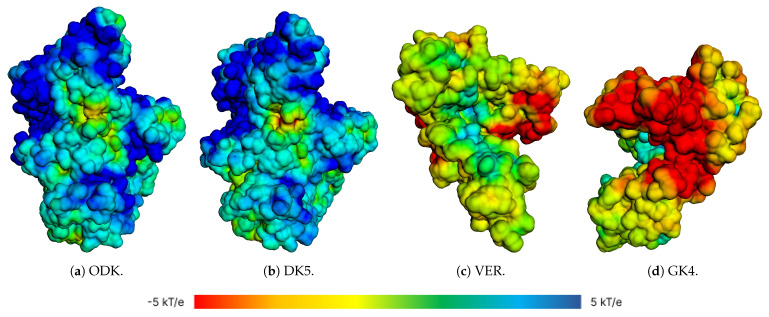
Electrostatic potential of (**a**) ODK-PARP−1, (**b**) DK5-PARP−1, (**c**) VER-YAP1, (**d**) GK4-YAP−1. Central image represents the overall electrostatic potential map.

By performing the analysis of the APBS results in the structural conformations of the proteins for both PARP-1 and YAP-1 interacting with the two best molecules in each case, we can quickly observe that, in the case of PARP-1, the electrostatic potential hypersurface is positive and neutral in some regions, due to the nature of the residues present on the surface of the protein. In contrast, for the case of YAP-1, the potential hypersurface is shown to be negative and neutral in some regions due to the residues in the surface section of the protein (see Figure 12).

**Figure 13:**
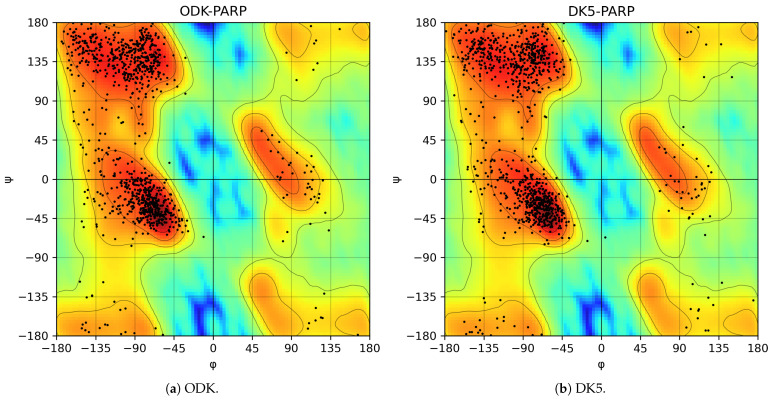
Ramachandran diagram analysis of (**a**) ODK and (**b**) DK5 in a rainbow scale: red indicates more accumulation, and blue indicates less accumulation of residuals.

Additionally, the Ramachandran analysis was performed for the final interacting structures between PARP−1 and the ODK and DK5 molecules, where we obtained for the case of the interacting system with ODK (See Figure 13a), 84.4% of the residues were found in the favored regions, while 14.3% are in allowed areas and 1% are in the generously allowed regions, with only 0.3% being in the disfavored areas. We performed this analysis on the protein, excluding glycines, prolines, and terminal residues, that is, 897 residues. In the case of DK5 (See Figure 13b), we found 85.6% in the favored regions, 13.3% in the permitted areas, 0.7% in the generously allowed regions, and 0.4% in the disfavored areas, out of a total of 897 residues, which excluded glycines, prolines, and terminal residues.

**Figure 14:**
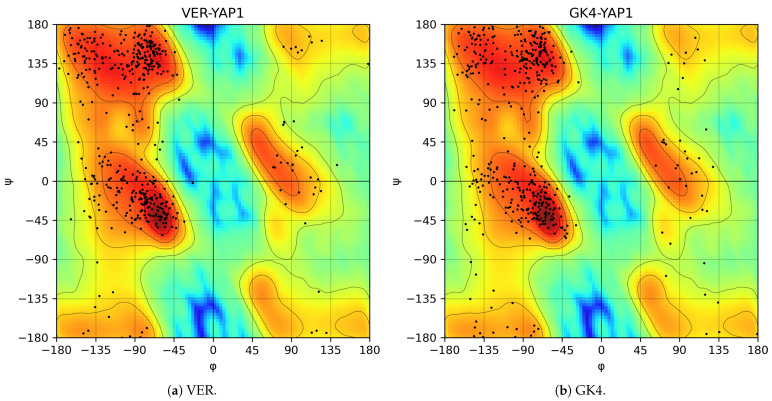
Ramachandran diagram analysis of (**a**) VER and (**b**) GK4 in a rainbow scale: red indicates more accumulation, and blue indicates less accumulation of residuals.

When we analyze Ramachandran’s results for the YAP-1 systems and the VER and GK4 molecules, in the case of the VER molecule (see Figure 14a), we find that 76.7% of the residues were in the favored regions, 18.4% were in the allowed areas, 3.7% were in the generously allowed regions, and 1.2% were in the disfavored areas, out of a total of 407 residues, in which we did not consider glycines, prolines, and terminal residues. Likewise, for the case of GK4, we found that 74.0% of residues were in the favored regions, 21.4% in the allowed areas, 2.9% in generously allowed regions, and 1.7% in disfavored regions of a total of 407 residues, where glycines, prolines, and terminal residues have not been considered (see Figure 14b).

When performing the ADMET analysis for molecular systems regarding compounds targeting the PARP1 protein (See Table 5), MC1 stands out on its excellent solubility profile (logS of −1.22) and high intestinal permeability, suggesting good bioavailability. In addition, it has low toxicity, making it a viable candidate. DK5 also shows good permeability, although it has a smaller volume of distribution, which may limit its efficacy in peripheral tissues. Although ODK and VDK have low solubility, they present relatively broad distribution profiles. Still, their toxicity is worrying, with risks of mutagenicity (positive Ames) and skin sensitization in the case of ODK. Both compounds also show inhibitory activity on CYP450, which could increase the risk of drug interactions. GK4, in addition to its activity on YAP−1, shows potential as a PARP1 inhibitor, with sound absorption and an acceptable toxicity profile. Finally, MC2 raises concerns regarding hepatotoxicity.

Compounds targeting the YAP−1 protein show varied ADMET profiles (see Table 5), with GK1 and GK4 standing out for their most favorable properties. GK1 has good solubility (logS of −1.588), high intestinal permeability, and low toxicity, making it a good candidate for development as a YAP−1 inhibitor. GK4, although having slightly lower solubility than GK1, maintains an acceptable absorption and distribution profile with low toxicity. In contrast, GK2 shows low intestinal absorption (HIA of 0.116) and lower permeability, which could limit its efficacy in clinical applications. Despite having good intestinal permeability, GK3 is limited by its ability to block hERG channels, implying a risk of arrhythmias. MC2, although having a good absorption profile, presents a high risk of liver toxicity (positive DILI), which could limit its therapeutic use. Finally, VER has mixed ADMET properties with good solubility and permeability. It also presents a risk of hERG inhibition and hepatotoxicity, suggesting that optimizations would be necessary to improve its safety.

## 4. Conclusions

Our results have led us to the following conclusions: the analysis shows that in the case of PARP−1 the naturally occurring molecule N-(3-Methoxybenzyl)-(9Z,12Z,15Z)-octadecatrienamide (DK5) is the most promising compared to Olaparib (ODK) which is a repositioned drug, this analysis is derived from the information that we were able to extract from the MMPBSA calculations. In contrast, the naturally occurring molecule for YAP-1 is stearic acid (GK4) compared to the repositioned drug Verteporfin (VER), as in the case of PARP−1, it concerns the MMPBSA calculations of the free energies of interaction. From the ADMET analysis, we can indicate that ODK and DK5 present toxicity, while VER and GK4 do not present toxicity. It is essential to highlight that in the case of ODK, its free energy of interaction is less favorable than DK5 when we compare both energies; in the case of VER it is slightly more favored than GK4 in its free energy of interaction, which makes us conclude that the compounds DK5 and GK4 found in *Lepidium meyenii* (Peruvian Maca) could be potential drugs for used in the treatment of cancer involving glioblastoma multiforme. We hope some experimental group can conduct the corresponding experiments to validate our theoretical results.

## Figures and Tables

**Table 1:** Analysis of druggable pockets of PARP-1 and YAP-1 proteins.

Protein	Pocket	Surface Area (${Å}^{2}$)	Volume (${Å}^{3}$)	DrugScore	Druggability
PARP-1	1	7220.25	14,216.62	8047.00	Strong
	2	6021.25	10,959.88	5759.00	Strong
	3	4917.75	9253.25	5613.00	Strong
	4	3259.75	6120.75	3390.00	Strong
	5	2481.25	4174.62	2678.00	Strong
	6	2584.75	5497.62	2651.00	Strong
YAP-1	1	1063.50	1767.75	2340.00	Strong
	2	1496.00	2576.00	2111.00	Strong
	3	370.75	532.50	284.00	Medium
	4	553.25	822.38	82.00	Medium
	5	456.50	592.62	−59.00	Medium
	6	715.75	856.62	−129.00	Medium

**Table 2:** Binding energies for each of the interacting systems.

Protein	Inhibitor	Energy (kcal/mol)
PARP-1	MC1	−8.10
	MC2	−8.73
	DK5	−8.99
	GK4	−6.76
	ODK	−11.13
	VDK	−9.34
YAP-1	GK1	−5.40
	GK2	−7.16
	GK3	−7.92
	GK4	−6.58
	MC2	−9.16
	VER	−10.40

**Table 3:** Binding energies from the MMPBSA-PARP1 analysis.

Energy Component (kcal/mol)	Poli-(ADP-Ribose)–Polimerase-1 (PARP1)					
	MC1	MC2	DK5	GK4	ODK	VDK
Van der Waals Energy	−48.92 ± 2.47	−55.00 ± 2.03	−53.89 ± 2.55	−45.82 ± 2.22	−54.15 ± 2.31	−41.51 ± 2.33
Electrostatic Energy	−3.17 ± 3.30	0.13 ± 2.19	−0.31 ± 2.43	−3.37 ± 2.82	−9.91 ± 2.96	−1.74 ± 4.29
Polar Solvation Energy	18.68 ± 3.07	29.11 ± 3.57	20.81 ± 3.07	20.27 ± 4.10	25.45 ± 2.19	16.89 ± 4.56
SASA Energy	−5.24 ± 0.26	−5.65 ± 0.20	−5.81 ± 0.25	−5.43 ± 0.24	−5.15 ± 0.20	−3.76 ± 0.17
SAV Energy	−50.75 ± 5.48	−53.37 ± 5.63	−54.72 ± 5.35	−49.83 ± 5.06	−47.34 ± 5.70	−37.99 ± 3.08
Binding energy	−89.39 ± 7.20	−84.74 ± 7.31	−93.89 ± 6.79	−84.16 ± 6.42	−91.06 ± 6.95	−68.15 ± 6.74

**Table 4:** Binding energies from MMPBSA-YAP1 analysis.

Energy Component (kcal/mol)	Yes1 (Associated Transcriptional Regulator YAP-1)					
	GK1	GK2	GK3	GK4	MC2	VER
Van der Waals Energy	−31.33 ± 4.04	−35.55 ± 4.08	−47.62 ± 4.62	−50.62 ± 2.53	−55.40 ± 2.55	−64.28 ± 4.50
Electrostatic Energy	−3.14 ± 4.41	−0.95 ± 2.70	−5.26 ± 2.97	−4.18 ± 2.20	−3.22 ± 2.69	−9.54 ± 4.75
Polar Solvation Energy	10.23 ± 2.40	17.46 ± 3.23	24.67 ± 3.82	16.90 ± 3.06	26.18 ± 4.48	24.57 ± 2.35
SASA Energy	−3.56 ± 0.27	−4.84 ± 0.39	−5.89 ± 0.28	−5.47 ± 0.22	−5.91 ± 0.24	−7.53 ± 0.41
SAV Energy	−34.55 ± 4.92	−43.58 ± 7.03	−54.32 ± 6.34	−57.40 ± 4.47	−58.91 ± 4.92	−64.59 ± 7.26
Binding energy	−62.36 ± 10.28	−67.47 ± 9.84	−88.42 ± 10.86	−100.77 ± 6.50	−97.29 ± 7.18	−121.37 ± 10.75

**Table 5:** ADMET prediction of the five nicotine analogs obtained using the ADMETlab 3.0 server.

Property	Model Name	GK1	GK2	GK3	GK4	MC2	VER	MC1	DK5	ODK	VDK
Physicochemical	logS	−1.59	−3.70	−0.85	−0.85	−0.87	−3.12	−1.22	−4.50	−3.05	−3.2
	logP	2.558	3.197	1.699	1.674	2.308	2.712	2.389	3.112	3.009	2.844
	logD	0.900	1.000	0.682	0.944	0.52	0.865	0.659	1.312	1.112	0.998
	Molecular Weight (Da)	219.13	317.29	331.29	284.27	383.28	389.50	218.40	401.30	412.80	432.20
	QED	0.583	0.329	0.370	0.336	0.207	0.512	0.471	0.62	0.543	0.487
	H-Bond Donors	3	5	1	1	1	2	4	3	2	1
	H-Bond Acceptors	3	4	2	2	3	4	3	5	4	4
	Caco-2 Permeability	−5.25	−5.12	-	-	-	−4.90	−4.76	−4.79	−4.60	−4.78
	MDCK Permeability	−4.6	−4.8	-	-	-	−4.7	−4.9	−4.6	−4.8	−4.9
Absorption	Pgp Inhibitor	No	No	No	No	No	Yes	No	No	Yes	No
	Pgp Substrate	No	No	Yes	No	Yes	Yes	No	No	No	Yes
	HIA	0.957	0.116	0.682	0.944	0.520	0.821	0.719	0.850	0.753	0.678
Distribution	PPB	0.675	0.759	0.284	0.074	0.966	0.902	0.751	0.822	0.814	0.803
	VD	47.06	51.90	16.00	16.00	1.70	39.80	45.00	41.20	42.70	40.60
	Fu	0.001	0.329	0.370	0.336	0.207	0.258	0.150	0.112	0.302	0.290
	CYP1A2 Inhibitor	Yes	Yes	No	No	Yes	No	Yes	No	Yes	No
Metabolism	CYP2C19 Substrate	No	No	Yes	Yes	No	No	No	No	Yes	Yes
	CYP3A4 Inhibitor	Yes	Yes	No	Yes	Yes	Yes	No	No	Yes	No
	CL (L/h)	3.15	3.57	2.80	2.85	3.32	2.99	3.42	2.71	2.85	2.77
	t1/2 (h)	0.215	0.260	0.186	0.171	0.176	0.190	0.210	0.230	0.215	0.221
Excretion	hERG Blockers	No	No	Yes	No	Yes	Yes	No	No	Yes	No
	DILI	No	No	No	No	Yes	Yes	No	No	No	Yes
	Ames Toxicity	No	No	No	No	No	No	No	Yes	Yes	No
	Skin Sensitization	No	No	No	No	No	No	No	No	Yes	No

## Data Availability

Data are contained within the article.
